# Regular follow-up visits reduce the risk for asthma exacerbation requiring admission in Korean adults with asthma

**DOI:** 10.1186/s13223-018-0250-0

**Published:** 2018-07-10

**Authors:** Hye Jung Park, Min Kwang Byun, Hyung Jung Kim, Chul Min Ahn, Chin Kook Rhee, Kyungjoo Kim, Bo Yeon Kim, Hye Won Bae, Kwang-Ha Yoo

**Affiliations:** 1 Department of Internal Medicine, Gangnam Severance Hospital, Yonsei University College of Medicine, 211 Eonju-ro Gangnam-gu, Seoul, 135-720 South Korea; 2 Division of Pulmonary, Allergy and Critical Care Medicine, Department of Internal Medicine, Seoul St Mary’s Hospital, College of Medicine, The Catholic University of Korea, Seoul, South Korea; 3 Healthcare Review and Assessment Committee, Health Insurance Review & Assessment Service, Seoul, South Korea; 4 Division of Quality Assessment Management, Health Insurance Review & Assessment Service, Seoul, South Korea; 5 Division of Pulmonary, Allergy and Critical Care Medicine, Department of Internal Medicine, Konkuk University School of Medicine, Seoul, South Korea

**Keywords:** Asthma, Compliance, Exacerbation

## Abstract

**Background:**

Asthma requires regular follow-up visits and sustained medication use. Although several studies have reported the importance of adherence to medication and compliance with the treatment, none to date have reported the importance of regular follow-up visits. We investigated the effects of regular clinical visits on asthma exacerbation.

**Methods:**

We used claims data in the national medical insurance review system provided by the Health Insurance Review and Assessment Service of Korea. We included subjects aged ≥ 15 years with a diagnosis of asthma, and who were prescribed asthma-related medication, from July 2013 to June 2014. Regular visitors (frequent visitors) were defined as subjects who visited the hospital for follow-up of asthma three or more times per year.

**Results:**

Among 729,343 subjects, 496,560 (68.1%) were classified as regular visitors. Old age, male sex, lack of medical aid insurance, attendance of a tertiary hospital, a high Charlson comorbidity index, and a history of admission for exacerbated asthma in the previous year were significant determining factors for regular visitor status. When we adjusted for all these factors, frequent visitors showed a lower risk of asthma exacerbation requiring general ward admission (odds ratio [OR] 0.48; 95% confidence interval [CI] 0.47–0.50; *P* < 0.001), emergency room admission (OR 0.83; 95% CI 0.79–0.86; *P* < 0.001), and intensive care unit admission (OR 0.49; 95% CI 0.44–0.54; *P* < 0.001) than infrequent visitors.

**Conclusions:**

Regular clinical visits are significantly associated with a reduced risk of asthma exacerbation requiring hospital admission in Korean adults with asthma.

## Background

Asthma is characterized by chronic airway inflammation that requires regular followed-up visits and continuous medication use [1, 2]. Several studies have reported that a large proportion of asthma patients (10–60%) showed poor adherence to medication [3–5]. There are numerous reasons for such poor adherence. Some patients prefer to use medication for a shorter period of time, and therefore, they do not take medication when they are asymptomatic [6–8]. Other reasons include forgetfulness, inconvenience, and unawareness of the importance of medication [9]. However, many studies have revealed that subjects with good adherence to medication use were at reduced risk for asthma exacerbation and mortality as compared with subjects with poor adherence [10–12]. Therefore, clinicians have attempted to increase adherence to obtain a better prognosis by using inhaler reminders, good partnership, and intensive patient education and training [13, 14].

Regular clinical visits may have increased the opportunity to increase adherence and compliance. When patients visit clinics frequently, clinicians can pay careful attention to the patients, educate them in detail, judge the situation more appropriately, and prescribe the proper medication. Together, this might lead to an improved prognosis; however, to date, there are no data to prove this hypothesis. We therefore investigated the hypothesis that regular visits would have a protective effect against asthma exacerbation, by using a large body of claims data from the national medical insurance review system that covers most Korean individuals.

## Methods

### Ethics

This study was approved by the Ethics Committee of the National Evidence-Based Healthcare Collaborating Agency. The need for informed consent was waived by the institutional review board of the Gangnam Severance Hospital, Yonsei University Health System (Approval Number: 3-2016-0332).

### Data sources

Korea has adopted a single mandatory government-established health insurance system, and the Health Insurance Review and Assessment Service (HIRA) is an agency responsible for evaluating all medical claim data in Korea. As the HIRA has accumulated all medical records, most Korean citizens are thought to be covered by this system [15]. We retrospectively reviewed and analyzed the data from the HIRA database of the national medical insurance review system of Korea.

### Study populations

Patients with asthma were defined as follows, based on previous articles that had used HIRA data [15, 16]; this was also concordant with the definition used by HIRA for the asthma quality evaluation program. We included all patients aged ≥ 15 years, with asthma (J45.0, J45.1, J45.8, J45.9, J46.0, J46.1, J46.8, or J46.9 from the International Classification of Diseases-10th revision) as the principal or the first additional diagnosis, from July 2013 to June 2014. The included patients were prescribed asthma-related medication (inhaled, oral, or injected) on at least two outpatient visits, or on at least one outpatient visit along with systemic corticosteroids prescribed on admission. Asthma-related medications included corticosteroids, leukotriene antagonists, long-acting β2-agonists, short-acting β2-agonists, anticholinergics, and xanthine derivatives.

### Definition of terms

Regular visitors (frequent visitors) were defined as subjects who visited the hospital for clinical follow-up of asthma three or more times per year, regardless of the visit interval and visit site; alternatively, they were classified as infrequent visitors. The Charlson comorbidity index, which is a value facilitating prediction of prognosis and mortality based on comorbidities, was calculated as previously described [17, 18]. Asthma exacerbation requiring hospital admission was defined as an admission to a general ward, emergency room (ER), or intensive care unit (ICU), with a diagnosis of asthma as the principal or the first additional diagnosis.

### Statistical analysis

We used the *t* test and Chi square test to identify differences in continuous data and categorical variables between frequent and infrequent visitors, respectively. Univariate and multivariate analyses were used to find significant factors for regular visitor status and admission with asthma exacerbation, using logistic regression analysis. We used SPSS v18.0 (IBM Corp, Armonk, NY). A *P* < 0.05 was considered statistically significant.

## Results

### Demographics of subjects

We reviewed 729,343 subjects enrolled in National Health Insurance Database. The mean age of subjects was 57.2 years, and 40.3% were male. Most subjects (82.2%) were followed-up in a primary hospital, while others (21.7%) attended a tertiary hospital. Pulmonary function tests were performed in 21.2% of subjects. Although data was not shown, 11.1% of subjects attended more than two hospital types. Most of the subjects (66.2%) had allergic rhinitis. Hypertension, diabetes mellitus, and metabolic syndrome accompanied asthma in 15.3, 7.7, and 6.5% of patients, respectively. The mean Charlson comorbidity index was 1.3. Some subjects (2.6%) had experienced at least one additional asthma exacerbation requiring hospital admission in the previous year. A total of 68.1% of cases were regular visitors (Table 1).

**Table 1:** Demographics of subjects

Parameters	N (%)
Age (mean ± SD)	57.2 ± 17.9
Male	293,762 (40.3)
Subjects with medical aid insurance	675,479 (92.6)
Hospital type	
Primary	599,460 (82.2)
Secondary	56,484 (7.7)
Tertiary	158,043 (21.7)
Tests subjects were taken	
Chest X-rays	118,479 (16.2)
Chest CT	2291 (0.3)
Pulmonary function test	154,984 (21.2)
Co-morbidity	
Ischemic heart disease	21,191 (2.9)
Osteoporosis	16,138 (2.2)
Depressive disorder	7703 (1.1)
Arthritis	24,392 (3.3)
Diabetes mellitus	56,355 (7.7)
Pneumothorax	716 (0.1)
Congestive heart failure	10,914 (1.5)
Hypertension	111,249 (15.3)
Anemia	7412 (1.0)
Metabolic syndrome	47,605 (6.5)
Allergic rhinitis	482,540 (66.2)
Charlson’s comorbidity index (mean ± SD)	1.3 ± 0.7
Subjects admitted with exacerbated asthma in previous year	18,899 (2.6)
Frequent visitor	496,560 (68.1)
Total	729,343 (100.0)

*SD* standard deviation, *CT* computed tomography

### Significant factors for frequent visitors

Frequent visitors were significantly older. The percentage of male patients was slightly higher in the frequent visitor group, compared with the infrequent visitor group. They had less medical aid insurance and frequently attended a tertiary hospital. They also had a high Charlson’s comorbidity index, and had more frequently experienced admission with exacerbated asthma in the previous year, as compared to infrequent visitors (Table 2).

**Table 2:** Differences in patterns between infrequent and frequent visitors

Parameters	Infrequent visitor	Frequent visitor	*P* value
Age (mean ± SD)	52.9 ± 18.5	59.2 ± 17.5	< 0.001
Male	38.9%	40.9%	< 0.001
Medical aid insurance	95.1%	91.4%	< 0.001
Hospital type			< 0.001
Primary	81.3%	67.4%	
Secondary	4.6%	4.0%	
Tertiary	11.0%	13.8%	
Charlson’s comorbidity index (mean ± SD)	1.2 ± 0.5	1.4 ± 0.8	< 0.001
Admission with exacerbated asthma in previous year	1.0%	3.3%	< 0.001

*SD* standard deviation

We set out to define significant factors that determine frequent visitor status. Multivariate analysis confirmed that old age (odds ratio [OR] 10.5; 95% confidence interval [CI] 1.01–1.02; *P* < 0.001), male sex (OR 1.05; 95% CI 1.04–1.07; *P* < 0.001), tertiary hospital attendance (OR 4.97; 95% CI 4.84–5.10; *P* < 0.001), a higher Charlson comorbidity index (OR 1.40; 95% CI 1.39–1.42; *P* < 0.001), and a history of admission with exacerbated asthma in the previous year (OR 1.72; 95% CI 1.64–1.80; *P* < 0.001) were significant contributing factors to frequent visitor status. Subjects with medical aid insurance tended not to be frequent visitors (OR 0.69; 95% CI 0.68–0.71; *P* < 0.001) (Table 3).

**Table 3:** Significant factors contributing to frequent visitor status

Parameters	Univariate analysis		Multivariate analysis	
	OR (95% CI)	*P* value	OR (95% CI)	*P* value
Age	1.02 (1.02–1.02)	< 0.001	1.01 (1.01–1.02)	< 0.001
Male	1.09 (1.08–1.10)	< 0.001	1.05 (1.04–1.07)	< 0.001
Medical aid insurance	0.55 (0.54–0.56)	< 0.001	0.69 (0.68–0.71)	< 0.001
Hospital type				
Primary	0.82 (0.81–0.83)	< 0.001	3.95 (3.84–4.06)	< 0.001
Secondary	1.55 (1.52–1.58)	< 0.001	3.35 (3.25–3.45)	< 0.001
Tertiary	2.25 (2.22–2.28)	< 0.001	4.97 (4.84–5.10)	< 0.001
Charlson’s comorbidity index	1.71 (1.69–1.72)	< 0.001	1.40 (1.39–1.42)	< 0.001
Admission with exacerbated asthma in previous year	3.28 (3.14–3.42)	< 0.001	1.72 (1.64–1.80)	< 0.001

*OR* odds ratio, *CI* confidence interval

### Number of subjects with asthma exacerbation according to visit frequency

We compared unadjusted healthcare utilization with asthma exacerbation between infrequent and frequent visitors. Frequent visitors showed more frequent exacerbations requiring admission to the general ward (4.6% vs. 3.2%; *P* < 0.001), ER (2.8% vs. 1.2%; *P* < 0.00), or ICU (0.4% vs. 0.3%; *P* < 0.001) than did infrequent visitors (Table 4).

**Table 4:** Number of subjects with asthma exacerbations, according to visit frequency

Parameters	Number of subjects experienced asthma exacerbation in infrequent visitor (%)	Number of subjects experienced asthma exacerbation in frequent visitor (%)	*P* value
General ward admission	7504 (3.2)	22,697 (4.6)	< 0.001
ER admission	2881 (1.2)	13,876 (2.8)	< 0.001
ICU admission	596 (0.3)	1958 (0.4)	< 0.001
Total	232,783 (100.0)	496,560 (100.0)	

*ER* emergency room, *ICU* intensive care unit

### Regular visits are protective factors against asthma exacerbation

Concordant with the results shown in Table 3 in univariate analysis, frequent visitor status was a significant risk factor for asthma exacerbation requiring general ward admission (OR 1.44; 95% CI 1.40–1.48; *P* < 0.001), ER utilization (OR 2.29; 95% CI 2.20–2.39; *P* < 0.001), and ICU admission (OR 1.54; 95% CI 1.41–1.69; *P* < 0.001). However, we sought to define whether the frequent visits increased the risk for or were protective against asthma exacerbation in adjusted conditions. In multivariate analysis, in which we included all the factors significant for frequent visitor status, we found the opposite results, as follows. Old age, male sex, subjects without medical aid insurance, tertiary hospital type attendance, a higher Charlson comorbidity index, and admission with exacerbated asthma in the previous year were significant predictive factors for asthma exacerbation requiring admission. Moreover, we found that frequent hospital visits was factor protecting against asthma exacerbation requiring general ward admission (OR 0.48; 95% CI 0.47–0.50; *P* < 0.001), ER utilization (OR 0.83; 95% CI 0.79–0.86; *P* < 0.001), and ICU admission (OR 0.49; 95% CI 0.44–0.54; *P* < 0.001) (Table 5).

**Table 5:** Univariate and multivariate analyses for asthma exacerbation requiring admission

Parameters	Univariate analysis		Multivariate analysis	
	OR (95% CI)	*P* value	OR (95% CI)	*P* value
General ward admission with exacerbated asthma				
Age	1.04 (1.04–1.04)	< 0.001	1.02 (1.02–1.02)	< 0.001
Male	1.08 (1.06–1.11)	< 0.001	0.79 (0.77–0.81)	< 0.001
Medical aid insurance	0.43 (0.42–0.44)	< 0.001	0.81 (0.78–0.85)	< 0.001
Hospital type				
Primary	0.19 (0.19–0.20)	< 0.001	1.44 (1.39–1.48)	< 0.001
Secondary	7.75 (7.56–7.95)	< 0.001	15.97 (15.40–16.56)	< 0.001
Tertiary	12.25 (11.93–12.58)	< 0.001	20.14 (19.44–20.88)	< 0.001
Charlson’s comorbidity index	2.11 (2.09–2.13)	< 0.001	1.62 (1.60–1.64)	< 0.001
Admission with exacerbated asthma in previous year	14.16 (13.70–14.63)	< 0.001	4.39 (4.22–4.56)	< 0.001
Frequent visitor	1.44 (1.40–1.48)	< 0.001	0.48 (0.47–0.50)	< 0.001
ER admission with asthma exacerbation				
Age	1.02 (1.02–1.02)	< 0.001	0.99 (1.00–1.00)	0.023
Male	1.44 (1.40–1.48)	< 0.001	1.10 (1.07–1.14)	< 0.001
Medical aid insurance	0.48 (0.46–0.050)	< 0.001	0.79 (0.75–0.83)	< 0.001
Hospital type				
Primary	0.25 (0.25–0.26)	< 0.001	2.03 (1.96–2.11)	< 0.001
Secondary	2.93 (2.82–3.05)	< 0.001	4.10 (3.91–4.30)	< 0.001
Tertiary	60.61 (56.91–64.56)	< 0.001	77.05 (72.11–82.33)	< 0.001
Charlson’s comorbidity index	1.84 (1.82–1.86)	< 0.001	1.38 (1.36–1.40)	< 0.001
Admission with exacerbated asthma in previous year	9.51 (9.11–9.92)	< 0.001	2.55 (2.43–2.67)	< 0.001
Frequent visitor	2.29 (2.20–2.39)	< 0.001	0.83 (0.79–0.86)	< 0.001
ICU admission with asthma exacerbation				
Age	1.08 (1.08–1.08)	< 0.001	1.05 (1.05–1.06)	< 0.001
Male	1.55 (1.44–1.68)	< 0.001	1.16 (1.07–1.26)	< 0.001
Medical aid insurance	0.39 (0.35–0.43)	< 0.001	0.84 (0.75–0.94)	0.002
Hospital type				
Primary	0.19 (0.17–0.20)	< 0.001	1.42 (1.30–1.54)	< 0.001
Secondary	2.33 (2.10–2.59)	< 0.001	2.09 (1.87–2.34)	< 0.001
Tertiary	158.04 (121.81–205.04)	< 0.001	122.29 (93.79–159.45)	< 0.001
Charlson’s comorbidity index	2.13 (2.09–2.18)	< 0.001	1.51 (1.48–1.55)	< 0.001
Admission with exacerbated asthma in previous year	11.68 (10.64–12.82)	< 0.001	2.47 (2.23–2.72)	< 0.001
Frequent visitor	1.54 (1.41–1.69)	< 0.001	0.49 (0.44–0.54)	< 0.001

*OR* odds ratio, *CI* confidence interval, *ER* emergency room, *ICU* intensive care unit

## Discussion

This large retrospective population study of asthma patients defined significant decisive factors for regular hospital visitor status, and demonstrated that regular visits were significantly associated with a better asthma prognosis. Superficially, regular visits would seem to be associated with increased asthma exacerbation, as shown in the unadjusted data in Table 2. We first identified significant decisive factors for regular visitor status, although age and male sex are not clinically significant factors. Importantly, admission with exacerbated asthma in the “previous year” was a significant factor for frequent visitor status. Thus, asthma patients who suffered asthma exacerbation in the “previous year” may visit the hospital frequently. Further, we observed acute exacerbation in the “next year” in both frequent and infrequent visitors. After we controlled for the effect of asthma exacerbation in the “previous year,” including the effects of other significant decisive factors on regular visitor status, we found that the risk of acute exacerbation in the “next year” was reduced in frequent visitors. Regular visits (at least three or more times per year) were significantly associated with a reduction in the risk of general ward, ER, and ICU admission for exacerbated asthma by 52, 17, and 51%, respectively, as compared to infrequent hospital visits. This study therefore revealed that regular visits may have protective effects against asthma exacerbation. We therefore suggest that clinicians should encourage asthma patients to participate in regular and frequent follow-up visits in order to reduce the risk for asthma exacerbation requiring admission.

Asthma is a chronic airway disease that requires long-term follow-up and consistent care. It is advisable to encourage patients to attend regular follow-up visits as a first step toward achieving a good prognosis. Patients require education regarding an asthma action plan [19], inhaler technique [20], required changes of medication dose and frequency [21], and improvement of the patient-physician partnership [22], in order to improve adherence and compliance, so as to facilitate a good prognosis. Regular visits are a precondition for these factors; notably, regular visitors may receive careful attention, be educated closely, and be assured that their medication is adjusted appropriately. Thus, regular visits will lead to increased adherence and compliance, with associated improvements in prognosis.

As in many countries, the majority of asthma patients are managed at primary hospitals (82.2%) by general practitioners in Korea because the initial management of asthma can be satisfactorily carried out at a primary hospital. Moreover, the primary hospital is generally more accessible, implies lower cost, and greater convenience. Therefore, the role of the general practitioner at primary hospitals in encouraging asthma patients to participate in regular and frequent follow-up visits is important.

In Korea, an asthma quality evaluation program was launched in 2013, to improve the quality of life of asthma patients and achieve a good prognosis. The assessment parameters included the frequency of pulmonary function tests, the frequency of prescription of mandatory asthma medications (inhaled corticosteroids and/or anti-leukotriene modifiers) [23], the frequency of prescription of non-mandatory asthma medications (beta-agonist and/or oral corticosteroids), and regular visitor status, which was defined as subjects who visited hospitals for asthma three or more times per year. We therefore attempted to identify whether regular visits facilitated a good prognosis. We used the same definition as described in the asthma appropriateness assessment in Korea in this study.

The GINA 2016 guidelines recommend that patients should preferably be seen 1–3 months for step-up and step-down management after starting treatment, and thereafter every 3–12 months for maintenance [2]. However, there has been no clear evidence to date supporting the necessity of frequent and regular visits. The persistence rate for clinic visits is reported to be 65 and 40% after 3 and 6 months of medication initiation, respectively [24]. Our findings suggest that asthma patients should be followed-up at least every 3–4 months (at least three or more times per year), and that this may be an easy approach for achieving a favorable prognosis.

The strength of this study was that we included a large population, reflecting almost all adults with asthma in Korea. The national medical insurance review system covers nearly all Korean individuals, and therefore, the 729,343 subjects enrolled in this study might encompass all the asthma patients in Korea. This is also supported by a calculation based on an asthma prevalence of about 2% and the total population size (about 40 million) in South Korea [25]. Therefore, this indicates the trustworthiness of the data.

This study has some limitations. First, this is a cross-sectional observational study, not a cohort study. Therefore, we cannot firmly conclude that regular visits directly reduce asthma exacerbations. We cannot ascertain causality; instead, we suggest that regular visits are significantly associated with reduced asthma exacerbations. Second, we could not analyze the potential variables that affect the frequency of visits and prognosis due to the retrospective nature of this study. For instance, adherence to medication, the dose of the inhaler, socio-economic status, clinical characteristics, residential district, and weather may be influential factors [26, 27]. In this retrospective study, which used claim data from a national medical insurance review system, the available variables were extremely restricted. Third, we used an operational definition for asthma diagnosis and exacerbation. We could not use the results of pulmonary function tests, or review the clinical charts of patients. Moreover, the operational definition of “asthma exacerbation” used in this study may contain a small number of patients who were admitted to the hospital for other reasons. This operational definition may influence the results; however, the large number of subjects may overcome this bias. Fourth, we could not discriminate mild exacerbations that did not require admission. Outpatients with mild exacerbations may visit clinics more frequently, and this may be a confounding factor. However, a history of exacerbation in previous years, for which we adjusted in this study, will also help to overcome this bias. Fifth, we cannot ignore collider bias; medication analysis could be considered to reveal indirect effects of other variables on asthma exacerbations. Last, we did not stratify according to the number of visits. Further studies are needed to determine an optimal threshold for the number of visits.

## Conclusions

This retrospective study, based on a large study population, demonstrated that regular clinic visits are significantly associated with reducing the risk of asthma exacerbation requiring hospital admission by 20–50% in Korean adults with asthma. We recommend that clinicians encourage asthma patients to participate in regular medical visits to achieve a good prognosis.

## Abbreviations

HIRA: Health Insurance Review and Assessment Service
CI: confidence interval
ER: emergency room
ICU: intensive care unit
OR: odds ratio
